# Current News and Opinion

**Published:** 1888-04

**Authors:** 


### Current News and Opinion.

*Editor Independent Practitioner :—*

Seeing an interesting article in the INDEPENDENT PRACTITIONER a short time ago, by Dr. L. P. Haskell, concerning vulcanite and its effects, allow me to quote two interesting cases coming under my care, and which I still keep track of.

CASE 1. Lady of middle age, wearing a full upper and under set on red vulcanite. After wearing for about three months, a puffiness of the mucus membrane of the mouth, sour stomach and looseness of the bowels were present. The latter trouble increased greatly, and at the end of eight months evacuations from the number of fifteen to twenty a day were not uncommon. Such effects followed as to produce alarm, and being a college student at the time, and under Dr. Haskell's instructions, his ideas were followed out. A gold upper denture with rubber (brown) attachments, and a Watts metal lower denture were made, with the result of a rapid gain from the first week, and to-day the patient is well and strong.

CASE 2. Was similar, though the mucus membrane of the mouth was not so much involved, but the bowel trouble about the same. Metal plates were made as in case one, and as a result a perfect cure was effected.

Were these cases merely coincident, or due to a specific cause? When will we follow the teachings of experience and wisdom as given to us by one so capable as Dr. Haskell? H. L. BARNUM, M. D.

---

*Editor Independent Practitioner :—*

My attention has been attracted to a description in the March number of the INDEPENDENT PRACTITIONER of "A New and Simple Matrix," as suggested by Dr. Samuel B. Freeman, of Chicago, Illinois, and while I do not wish to question that the device is entirely original, so far as Dr. Freeman is concerned, I do feel it is but justice to say that this form of matrix was conceived and devised by me several years ago, and exhibited before a meeting of the Susquehannah Dental Association, held at Lock Haven, Pa., in May, 1886, to which fact such well-known practitioners as Dr. C. S. Beck, of Wilkesbarre, Dr. G. W. Klump, of Williamsport, and others can testify. To further convince you of this claim, I herewith enclose one of these matrices, which was made by me and has been in use since 1886, the only difference being that my matrices have been made of thin steel and phosphor-bronze. This form of matrix was also exhibited by me before the Pennsylvania State Dental Society, held at Cresson Springs, Pa., in July, 1886. WM. B. MILLER, D. D. S.,

Altoona, Pa.

---

*Editor Independent Practitioner :—*

In the report of the Ninth International Medical Congress, published in your journal for February, 1888, I notice on page 83, that Dr. Busch, Director of the Dental Institute of the Royal University of Berlin, exhibited some circular knives as his invention. As I invented circular knives twelve years ago, and after using them for a number of years, published a note on them in the *Boston Medical and Surgical Journal* for May 31, 1883, it is difficult to see how Dr. Busch can rightly claim them as original, except in the way that Dr Land has appropriated my investigations of gas furnaces and enamel fittings, published many years ago. W. H. ROLLINS,

250 Marlborough St., Boston, Mass.

---

### THE INFLUENCE OF PERIOSTEUM IN THE FORMATION AND REPAIR OF BONE.

Dr. William McEuen of Glasgow, who is regarded as an authority, not only in Great Britain but upon the continents of Europe and America, a surgeon of vast clinical experience, contributes to *Annals of Surgery* a valuable paper upon this subject. It consists of a series of propositions supported by many convincing cases. The periosteum has usually been considered the active agent in the reproduction of bone, the medulla also being capable of the same function. The author contends that the periosteum is not the prime factor in bone regeneration, but that the soft tissues enclosed in the bone play the most important part in its development and reproduction. He admits the importance of maintaining the close relationship of the bone and the vascular periosteum, but urges that when it is temporarily separated from the bone, or when a part of the bone is entirely denuded of its periosteum, it is still supplied with enough of blood from the interior to maintain its vitality for an indefinite period, or until the formation of a new periosteum. His propositions are as follows:

Proposition A. When periosteum has been detached from an extensive area of an adult, healthy bone, and replaced after the lapse of some hours, union between the bone and periosteum can take place without sloughing or observable augmentation ensuing.

Proposition B. The periosteum may be separated from the bone for a period of days by inflammatory products, after the withdrawal of which reunion between the bone and the periosteum may take place without necrosis ensuing; showing that the temporary separation of the periosteum, even as a patholgoical result, is not necessarily attended by death of the bone.

Proposition C. The periosteum covering a portion of bone may be completely destroyed or permanently removed, yet the denuded bone may not only retain its vitality, but may throw out cells which will cover it and form a new periosteum.

Proposition D. A portion of bone which has its continuity severed on all sides, and at the same time has had all its periosteum removed, is capable of living and growing.

Proposition E. Not only do detached portions of bone deprived of their periosteum live when reimplanted in their original position, but such portions are capable of living after transplantation. Parts of deeper layers of bone which have no periosteal connection have lived and grown.

Proposition F. The periosteum does not initiate the reproduction of bone.

Proposition G. Bone may be regenerated independently of the medulla, which may itself be reproduced.

Proposition H. The histo-genetic phenomena support the foregoing observations, showing that periosteum does not generate bone.

The author shows that bone is reproduced from the osteoblasts, which are found in the interior of bone, in the intermediate tissues, in the Haversian canals, and under some circumstances in the central cavity. The periosteum acts merely as a sheath, as a protecting limiting membrane through which the bone receives a part of its blood supply, the more important part being provided by the nutrient vessels of the bone itself. In the light of these propositions, the successful implantation of teeth may be readily accounted for, the presence of the desiccated pericemantal membrane not being essential, and serving only as a kind of sponge-graft in the meshes of which the first deposits are protected. The connection of the implanted tooth will not probably be through a pericemental membrane, but by a direct bony union, more or less perfect.

---

### ILLINOIS STATE DENTAL SOCIETY.

The twenty-fourth annual meeting will be held at Cairo, commencing Tuesday, May 8th, 1888, continuing four days.

### REPORTS, ESSAYS AND DISCUSSIONS.

Report of Committee on Dental Science and Literature, Dr. M. L. Hanaford, Rockford, Chairman.

Report of Committee on Dental Art and Inventions, Dr. W. T. Magill, Rock Island, Chairman.

Dental Morphology and the Etiology of Irregularities, Dr. John J. R Patrick, Belleville. Discussion opened by Dr. E. H. Angle, Minneapolis, Minn.

Dental Electrics, Dr. J. Rollo Knapp, New Orleans. Discussion opened by Dr. G. W. Whitfield, Evanston.

Some Main Points Touching the Conservative Treatment of Teeth Whose Pulps are Nearly or Quite Exposed, Dr. J. D. Moody, Mendota. Discussion opened by Dr. J. N. Crouse, Chicago.

What shall we do with Inflamed Pulps? Dr. W. A. Johnston, Peoria. Discussion opened by Dr. A. W. Harlan, Chicago.

Prosthetic Dentistry. Some Difficult Cases and their Treatment, Dr. L. P. Haskell, Chicago. Discussion opened by Dr. Edgar D. Swain, Chicago.

The Rationale of Constructing and Attaching Artificial Crowns to Natural Roots of Teeth, Dr. John J. R. Patrick, Belleville. Discussion opened by Dr. Henry J. M'Kellops, St. Louis.

Making and Tempering Instruments, Dr. J. Frank Marriner, Ottawa. Discussion opened by Dr. George H. Cushing, Chicago.

Amalgams, Dr. W. B. Ames, Chicago. Discussion opened by Charles R. Taylor, Streator.

### CLINICS—WEDNESDAY.

Dr Truman W. Brophy, Chicago. Approximal Gold Filling, Molar or Bicuspid, using his Continuous Band Matrix.

Dr. W. N. Morrison, St. Louis. Regulating Appliances, Jack Screws Secured by Thin Platinum Bands, Springs, Wedges, etc.

Dr. E. H. Angle, Minneapolis, will have Models and Appliances Representing his new Methods of Regulating.

Dr. John J. R. Patrick, Belleville. The means he uses for Regulating Teeth will be shown by Charts and a Large Model with Movable Teeth.

Dr. C. A. Kitchen, Rockford. Tin and Gold Filling.

Dr. T. L. Gilmer, Quincy. Telescopic Platinum and Gold Crown.

Dr. J. Austin Dunn, Chicago. Medicinal Syringes.

Dr. James W. Cormany, Mt. Carroll. Gold Filling, using Electric Mallet.

Dr. A. E. Matteson, Chicago. Odds and Ends of Office Practice.

Dr. D. B. Freeman, Chicago. Labio Cervical Filling, using his Double Loop Clamp.

Dr. W. H. Taggart, Freeport. Corundum Point and Disk Maker.

THURSDAY.

Dr. J. Rollo Knapp, New Orleans, will have a very interesting Clinic on Crown and Bridge-work.

Dr. A. W. Harlan, Chicago. Pyorrhœa Alveolaris; Etiology, Prognosis and Treatment.

Dr. John J. R. Patrick, Belleville, will make and mount a Gold Crown for a member, and will have his Gold Crown Apparatus and Outfit Complete.

Dr. J. G. Reid, Chicago. Tin and Gold Approximal Filling.

Dr. H. H. Townsend, Pontiac. Approximal Gold Filling.

Dr. E. A. Wooley, Chicago. Root Canal Dryer.

Dr. K. B. Davis, Springfield. Gold Filling.

Dr. G. W. Whitfield, Evanston, will demonstrate Electrical Conditions Caused by Different Metals used in Filling Teeth.

Dr. E. D. Swain, Chicago. Gold Filling, Approximal.

Dr. C. N. Pruyn, Chicago. Cocaine in Minor Surgery and Extracting.

RAILROAD RATES.

Satisfactory rates have been obtained on all roads on the certificate plan. On the certificate plan the passenger pays full fare in going to the meeting, and secures his certificate thereof of the agent by requesting same at the time of purchase, and this certificate, when countersigned by the proper officials at the meeting, becomes authority for the sale of a return ticket over the same road between the same points at one-third fare, thus making one fare and one-third for the round trip.

HOTEL RATES.

| | |
|---|---|
| Halliday House | $2.50 to $3.00 |
| European | 1.50 |
| Arlington | 1.50 |
| Waverly | 1.00 |

The State Board of Dental Examiners will be at the Halliday House on Monday, May 7th, at 10 A. M. All candidates for examinations must be present at that hour. Examinations will last three days. C. R. E. KOCH, Sec'y.

### CONNECTICUT VALLEY DENTAL SOCIETY AND MASSACHUSETTS DENTAL SOCIETY.

The Connecticut Valley Dental Society and the Massachusetts Dental Society will hold a Union Meeting in Boston on the 10th, 11th, 12th and 13th of July next, at the Institute of Technology. All the Dental Societies in New England will be invited to unite with them, so that the meeting promises to be the largest ever held in this part of the country. The programmes will be sent out by the last of June. The work of the meeting will consist of essays, clinics and demonstrations in Dental Technics, and the presentation of inventions and improvements by members of the profession. Essays and papers will be given on subjects of practical and theoretical importance. Clinics will be given by prominent members of the profession. Clinics will in all cases be limited to actual operations with the patient in the chair. Under Dental Technics, will be shown methods of manipulation—processes not requiring the presence of patients—preparation of materials and making of instruments by members of the profession. The size of the meeting will offer a good opportunity to present appliances or new inventions. Those at a distance can send such with a brief description, and members will be appointed to present them at the meeting.

A full report of the meeting will be published in the professional journals. Members of the profession and journals are requested to kindly extend this notice as far as possible. Those having matters of interest under any of the above heads are invited to bring them to the attention of the Secretaries of the different committees, as given below. In connection with the meeting will be held an EXHIBITION modeled after the "Medical and Surgical Exhibition of the International Medical Congress." Recognizing the connection of the dental profession with the arts and sciences, all persons having articles, instruments or materials for use in dentistry, or that can be made of use in any way, are cordially invited to exhibit them. A large hall will be used for this purpose, and no charge will be made for space. It will, however, be necessary for exhibitors who desire to show apparatus requiring water or gas, to make their own arrangements with the janitor or treasurer of the hall. The exhibition of motors will be a prominent feature. The name and address of the exhibitors, with one line, descriptive of their exhibits, will be printed on the programme. Members of the profession knowing of manufacturers or dealers in new or interesting articles, are requested to send notice to the Secretary of Committee on Exhibits.

Secretary of Committee on Essays,
Dr. A. H. Gilson, 10 Temple Pl., Boston.

Secretary of Committee on Clinics,
Dr. E. C. Leach, 422 Col. Ave., Boston.

Secretary of Committee on Exhibits,
Dr. W. E. Page, Studio B'ld'g, Boston.

Secretary of Committee on Motors,
Dr. S G. Stevens, Evans House, Boston.

Please reserve the above dates on your appointment book.

G. F. Eames, M. D,, D. D. S.,
62 Trinity Terrace, Boston, Mass.,
Secretary Mass. Dental Society.

Geo. A. Maxfield, D. D. S.,

Holyoke Mass.,

Secretary Conn. Val. Dent'l Soc.

### NEW YORK COLLEGE OF DENTISTRY.

The twenty-second annual commencement of the New York College of Dentistry was held Saturday evening, March 10th, at Chickering Hall. Vice-President, Dr. Wm. T. LaRoche, presided. Valedictory address by Albert Westlake, Jr. Address to graduates by the Rev. Thomas Gallaudet, D. D.

Number of matriculates for the year, 211. Number receiving the degree of Doctor of Dental Surgery, 72, as follows:

### GRADUATES.

Franklin Porferio Arango, N. Y.
Vincent Washington Baker, N. J.
Charles Leslie Babcock, Ill.
Jacob Bate, Eng.
Herman Tobias Braun, Fla.
Francis Anthony Chicherio, N. Y.
Johannes Fredrich Wilhelm Clasing, Ger.
Joao Rodriguiz Da Silva, Demerara, B.G.
William Billings Drake Davenport, Mass.
William Salsbery Depew, N. Y.
David Nathan Feigensohn, Russia.
Edward Beardsby Griffith, Conn.
John Conrad Graft, N. J.
Fred Miner Hayward, Vt.
Jacob Hassinger, N. Y.
William Phillip Ives, Conn.
Edward Max Kettig, Ky.
Isaac Lyon, N. Y.
Cortez Jefferson Mapp, Ga.
Charles Everett Maine, Conn.
Nelson Merwin, N. Y.
Vincent Maurice Munier, N. Y.
Henry John Moore, Eng.
Horace Wilson Northrop, Conn.
Albert Brown Osmun, N. J.
Charles Albert Pickhardt, Conn.
Herman T. H. Russell, Nassau, N. P.
Arthur Percy Sturridge, Jamaica, W. I
Clarence Boice Stelle, N. J.
John Scott Sanger, N. Y.
Lewis Mapes Slocum, Jr., N. Y.
Edmund Louis Stevens, N. Y
Willard Forrest Tooker, N. Y.
Charles Frederick Weber, N. Y.
Harry Prescott Wilcox, Conn.
Alfred Wagner, N. Y.

William Fletcher Acton, Conn.
Winfield Hart Baldwin, Ill.
Virgilio Bazan, Cuba.
Stephen Edward Best, N. Y.
John L. Crater, N. J.
Julian Hyde Clark, N. Y.
José Anselmo Arcelio De Castro, Cuba.
William Lewis Drummond, N. Y.
Frank Morseman Dunn, N. Y.
Frederick Hubert Eichhorn, N. J.
Edward Fox, Ireland.
Walter Ephram Gerrish, Mass.
Karl Ferdinand Alfred Hane, Ger.
George Buck Herbert, N. J.
Elias Scudder Hall, N. J.
Henry Arthur King, Can.
Dennis Frank Keefe, Mass.
Charles Melzar Lindsay, Cal.
Simon Theodor Alfons Müller, Ger.
Frederick Louis Marshall, N. Y.
Eugene Walton Marshall, N. Y.
Louis Phillipe Margron, France.
John James Marchant, Brazil.
Frederick Nies, N. Y.
William Heston Pruden, N. J.
Edward Stevens Rugg, N. Y.
David Barclay Smith, N. J.
William Henry Steeves, N. B.
Lem Andrew Smith, N. Y.
Charles Simon Sweedy, Ill.
Livingston Andrew Snyder, Penn.
William James Taylor, N. Y.
Frank Van Blarcom, N. J.
Ernest Ford Weed, N. Y.
Albert Westlake, Jr., N. J.
Charles Dutton Wright, Can.

### OHIO COLLEGE OF DENTAL SURGERY.

The Forty-second Annual Commencement of the Ohio College of Dental Surgery (Dental Department of the University of Cincinnati), was held at College Hall, Cincinnati, Ohio, Wednesday, March 7, 1888.

GRADUATES:

| | | | |
|---|---|---|---|
| D. S. Anderson | Ohio. | Edwin Waddel | Ohio. |
| H. J. Bosart | " | W. W. Wallace | " |
| E. D. Broadwell | " | N. B. Hartwell | Indiana. |
| H. W. Cleland | " | M. A. Menges | " |
| D. M. Clement | " | B. C. Reid | " |
| Mrs. Jessie Dillon | " | E. J. Ward | " |
| M. H. Evans | " | J. W. Cartmell | Kentucky. |
| A. B. Fletcher | " | C. B. Clark | " |
| H. E. Harlan | " | J. F. Rees | " |
| F. Y. Herbert | " | W. C. Shankland | " |
| J. W. Hillman | " | O. T. Hanson | Illinois. |
| E. D. Hinkley | " | O. S. Mills | " |
| C. B. Hussey | " | A. H. Rainey | " |
| I. F. Hussey | " | B. L. Shobe | Kansas. |
| C. G. Lockwood | " | R. H. Updegraff | " |
| H. H. Robinson | " | J. A. Henning | Missouri. |
| C. A. Schuchardt | " | W. E. Scott | " |
| J. B. Schunck | " | W. E. Gochenour | Wisconsin. |
| H. T. Smith | " | R. D. Rood | " |
| Mrs. Z. V. Swift | " | W. A. Windell | Canada. |
| T. D. St. John | " | J. F. Hardman | Iowa |
| J. P. Tudor | " | R. B. Foster | Minnesota. |
| S. M. Ulrey | " | T. H. Sexton | Pennsylvania. |

---

### MINNESOTA HOSPITAL COLLEGE.

The Seventh Annual Commencement of the Dental Department of the Minnesota Hospital College was held in conjunction with the Medical Department, in the Hennepin Avenue M. E. Church, on Friday, March 16, 1888.

The address was delivered to the graduates by the Rev. Dr. D. J. Burrell, and the valedictory on behalf of the class by C, D. Snow, D. D. S.

The number of matriculates for the session was thirty-eight.

The degree of Doctor of Dental Surgery was conferred on the following graduates by C. H. Hunter, A. M., M. D., President of the Faculty:

| | |
|---|---|
| H. G. Dampier | Minnesota. |
| A. N. Cheney | Minnesota. |
| C. D. Snow | Minnesota. |
| H. T. Burnette | Minnesota. |
| C. L Sargent | Wisconsin. |
| D. H. Carpenter | Minnesota. |
| A. H. Benson, M. D | Wisconsin. |
| J. D. Jewett | Minnesota. |

### KANSAS CITY MEDICAL AND DENTAL COLLEGE.

The commencement exercises were held in Music Hall, Kansas City, Tuesday, March 13, 1888. The following graduates in dentistry received their diplomas: R. V. Anderson, J L. Leavel, F. L. Murdock, J. L. Reavis, W. L. Reed, H. S. Smith, E S. Sweet, C. M. Tindall.

### FIFTH DISTRICT DENTAL SOCIETY.

The Fifth District Dental Society of the State of New York will hold its twentieth annual meeting at Utica, Tuesday and Wednesday, April 10th and 11th, 1888. The session will be called to order at 2 P. M., at the Butterfield House. Applications for membership in the Society should be made on or before the day of meeting, to the Chairman of the Board of Censors, or to the Recording Secretary. The Board of Censors will be in attendance to examine candidates for admission to the Society. Members of the profession from other societies are cordially invited to be present and take part in the discussions.

C. J. PETERS, Syracuse, Rec. Sec'y

### SEVENTH DISTRICT DENTAL SOCIETY.

The twentieth annual convention will be held in Rochester, N. Y., April 24th and 25th. This Society includes the counties of Monroe, Wayne, Cayuga, Seneca, Yates, Ontario, Livingston and Steuben. Dentists practicing in any of these counties, and who wish to become members of the Society, can obtain information regarding requirements for membership, by writing to the Recording Secretary.

The convention will be made as interesting as possible. Members of the profession are invited to be present.

CHAS. T. HOWARD,

Recording Secretary.

224 E. Main St., Rochester, N. Y.

### EIGHTH DISTRICT DENTAL SOCIETY.

The twentieth annual meeting will be held in the lecture-room of the Society of Natural Sciences, Library Building, in the City of Buffalo, on Tuesday and Wednesday, April 17 and 18, 1888. All practicing dentists are cordially invited to be present.

S A. FREEMAN, Secretary.

### SOUTHERN ILLINOIS DENTAL SOCIETY.

The Southern Illinois Dental Society will hold its second annual meeting at Sadler's Opera House, Centralia, Ill, commencing Tuesday, April 10, 1888, at 10 A. M. Clinics will be a prominent feature of the programme. Papers and addresses by well-known dentists will be presented, and it is expected that an unusually profitable meeting will result.

G. W. ENTSMINGER, Secretary.

C. B. Rohland, T. W. Pritchett, A. D. Finch. } *Ex. Committee.*

### TEXAS DENTAL ASSOCIATION.

The next meeting of the Texas Dental Association will be held in Dallas, Tex., commencing on the first Tuesday in May, and continuing for four days.

T. H. LIPSCOMB, D. D. S., Cor. Sec'y.

### ILLINOIS STATE BOARD OF DENTAL EXAMINERS.

A meeting of the Board will be held at the St. Charles Hotel, Cairo, Ill., on Monday, the 14th day of May, at 11 o'clock A. M., and continue in session for three days. All parties desiring to obtain license to practice in this State, or having other business that requires the action of the Board, will please govern themselves accordingly. CHARLES R. E. KOCH, Secretary.

---

### KANSAS STATE DENTAL ASSOCIATION.

The seventeenth annual meeting will be held at Topeka, commencing Tuesday, April 24, 1888, and will continue four days. An unusually attractive programme will be presented, and it is expected that several gentlemen of prominence from abroad will be present and address the meeting.

C. B. GUNN, Secretary.

---

### GANGRENOUS TOOTH PULPS.

Dr. W. D Miller contributed to the Mississippi Valley Dental Association a paper on "Gangrenous Tooth Pulps as Centers of Infection." The paper does not pretend to present the subject as complete, the experiments being but little more than begun.

A tooth containing a gangrenous or putrefying pulp was extracted, mechanically cleaned and then brought for a moment into $\frac{5}{1000}$ solution of bichloride of mercury. It was rinsed with sterilized water to remove the sublimate, split with sterilized forceps and the putrid pulp removed with a sterilized needle. The material was then used to inoculate mice subcutaneously. One hundred and eighteen infections had been made, in a great majority of the cases followed by inflammation and swelling within twenty-four hours. At the end of the second or third day an abscess was usually found, containing pus densely impregnated with micro-organisms; some of the pulps developed more violent symptoms than others. In one case, at the end of the second day, a tumor developed containing a considerable quantity of fetid pus, with gases. A second mouse was inoculated with this pus, and a third from this one, and so on to the twelfth inoculation, each exhibiting the same characteristics, when the mouse died and ended the experiments.

In some cases there was blood poisoning. From these experiments, Dr. Miller believes that a putrid tooth-pulp may be a center of infection, or it may serve as a channel through which pathogenic bacteria from the oral cavity may invade the tissues surrounding the point of the root, or even obtain entrance into the circulation.

---

### CORRECTION.

In my work "Irregularities of the Teeth," on pages 157 and 158, certain retaining appliances are spoken of as having been devised by Dr Magill. Since the publication of the book the author has discovered that he was in error, and that the retainers referred to were designed by Dr. Guilford, and should have been credited to him. EUGENE S. TALBOT.

The following is a statement of the number of medical students attending the fifteen medical schools which existed in the United States in 1826 :

| | |
|---|---|
| University of Pennsylvania | 480 |
| College of Physicians and Surgeons of New York | 196 |
| Harvard College | 130 |
| Dartmouth College | 80 |
| University of Maryland | 215 |
| College of Physicians and Surgeons of the Western Dist. State of New York | 120 |
| Yale College | 82 |
| Medical College of Ohio | 22 |
| Vermont Academy of Medicine | 124 |
| Transylvania University | 235 |
| Medical School of Maine | 60 |
| Brown University | 40 |
| University of Vermont | 42 |
| Berkshire Medical School | 94 |
| Medical College of South Carolina | 50 |
| Total | 1970 |

—*Lancet-Clinic.*

---

The Medical and Surgical Reporter quotes from *The Lancet* as follows : In cases of catarrh of the antrum, Dr. Schiffers, of Liege, instead of extracting the second molar, gains access to the cavity through the opening in the middle meatus of the noses. Through this he inserts a director, and with the help of a curved, probe-pointed bistoury, he opens up a passage for the free exit of the confined secretion By the use of cocaine the patient suffers but little during the operation. Dr. Schiffers points out that catarrh of the antrum is frequently overlooked and mistaken for an affection of the mucus membrane of the nose. When an abundant fetid discharge runs from the nose, especially if it is intermittent, the existence of disease of the antrum should be suspected; a careful search should then be made, with the help of the nasal speculum and a good light, for the welling up of the secretion through the foramen in the middle meatus.

---

We incidentally criticised the grammatical construction of the following sentence, found in *The Western Dental Journal:*

"The school-boy essayists are catching fits, and right they should"—whereupon the "Miscellany" editor makes this rejoinder :

"Does the editor of the Independent Practitioner intend to pose before the profession as one of the 'literati,' or is he going to abandon dentistry for the broader field of inculcating correct grammar and pure English ?"

We are not "posing" at all, Deah Boy—we are not a poser; but do you not think that an idea may be quite as well expressed in good English as in bad; or do you believe that a knowledge of grammar is incompatible with the practice of dentistry ? And while you *do* have your hand in, pray will you not analyze the phrase, "A field of inculcating ?"

"If you want something pleasant to think of when you're in a dentist's chair, and he is fishing down into the root of one of your teeth for the end of a nerve, think of this; that it is only a few years since the only dentists were the regular physicians, and all they knew about dentistry was to throw a hideous kind of a steel grappling hook around a tooth and then haul away until something came. Now a dentist has to be a thorough chemist, a practical metallurgist, a good physician, an anatomist, a surgeon, a vulcanized rubber worker, to some extent an electrician, and have some feeling for the suffering of the people who come under his care. That's the way dental science has developed in a quarter of a century."—*New York Commercial Advertiser.*

---

The Annals of Surgery, which has just entered upon its fourth year, under the editorial management of Dr. L. S. Pilcher, of Brooklyn, and Dr C. B. Keetley, of London, Eng., is the only periodical in the English language that is devoted exclusively to surgery. All other departments of medicine are represented by a number of journals, but surgery has this alone, and we are not sure that it needs another. Each number contains a great variety of matter—exclusively original articles, editorials and summaries of surgical progress. *Annals of Surgery* is published monthly by J. H. Chambers, of St. Louis, at five dollars per annum. We can most heartily commend it to every one who has any interest whatever in surgical practice.

---

Dr. A. O. Hunt, of Iowa City, met with a painful accident lately, by falling and dislocating his shoulder. Pearson condoles with him, and says that it was perfectly right and proper that a man occupying his position should choose the time and place when he wishes to have his anatomy demoralized, for in these times of College Commencements and other riotous proceedings, a man is liable to *fall* in the most unexpected and inconvenient places, and have his bony structure handled by outsiders who do not know a clavicle from a cocktail, nor which to put in place first. But when the fall occurs amid home influences, his friends are spared the disagreeable task of explaining how the banana peel happened to be there.

---

The British Medical Journal for November 12, 1887, contains a paper by J. Hutchinson, Jr., upon the teeth in inherited syphilis. *Archives of Pediatrics* says that the whole subject is very carefully gone over. It was pointed out to be a fallacy to look for characteristic signs in the temporary teeth. They are, however, liable to premature decay and falling out. Of the permanent teeth, those first ossified—the incisors and the first molars—were the ones which showed to the greatest degree syphilitic deformity, the upper central incisors being the "test-teeth." Many syphilitic children have teeth normally formed.

---

Dr A. W. Harlan, of Chicago, read a paper before the Odontological Society of Great Britain, at its February meeting upon "The Management of Pulpless Teeth from the Standpoint of Daily Practice." At the same meeting Mr. Bland Sutton, F. R. C. S., read a paper upon "A Remarkable Case of Odontomes in a Thar, (Himalayan Goat.)

Dr. W. Storer How, in a paper read before the Mississippi Valley Dental Association, asserts that the great progress of American dentistry has been largely due to dental patents.

Ah, indeed! Of course the more important the patent and the greater its influence upon dentists and dentistry, the more has it contributed to this wonderful advance. Shall we then elevate Josiah Bacon and Dr. Sheffield to a position as the patron saints of dentistry, dethroning Harris, Parmley, Bond, Brown and their compeers, as the opponents of progress, because they did not advocate patents?

---

Dr. J. Austin Dunn has made a decided improvement in the needles of hypodermic syringes, and one which is especially adapted to the points of his medicinal syringe. Instead of soldering them to the hub, they are, by means of a rubber washer, adjusted as a nerve broach is adjusted in a screw holder. If the needle becomes stopped, it can easily be removed and heated without danger of unsoldering, or it may be thrown aside and a new piece of hollow wire inserted in its place. We do not see how the Dunn Syringe could be further improved.

---

"A Christian Science" professor in Chicago tried for seven days to cure an alveolar abscess, but the lady's face continuing to swell and the pain growing more severe, she resorted to a dentist who relieved the ache by extracting a molar. Seven dollars were willingly paid the scientist for humbugging, and the fifty cents charge of the dentist was grumbled at.—*Medical Standard.*

---

General A. W. Greely, Chief Signal Officer of the United States, contributes to the April *Scribner's* a valuable and timely article, answering the question "Where Shall We Spend Our Summer?" He gives some pertinent advice as to the best time for taking a short vacation, and explains the conditions of climate which make some resorts preferable to others.

---

A brass or copper surface, as a door plate or bell, may be silver-plated by rubbing with a soft piece of leather covered with the following preparation. One part of chloride of silver, three parts pearl ash, one and one-half parts common salt, and one part whiting. After the plating, the metal should be washed with a weak solution of soda and wiped dry.

---

Dr. Dresch, of Foix, France, says that the following method causes an immediate cessation of hiccough. The sufferer should close his external auditory canals by inserting a finger in each ear, exerting a certain degree of pressure; at the same time he should drink a few sips of any liquid, the glass or cup being held to his lips by another person.

---

The great telescope of the Lick observatory, in California, has been mounted upon a hollow iron pier thirty feet high. So marvelous is the magnifying power that the placing of a man's hand upon the iron pier causes expansion sufficient to throw a star entirely out of the field. The pier will have to be changed.

THE AMERICAN MEDICAL ASSOCIATION will hold its thirty-ninth annual session in Cincinnati, Ohio, on Tuesday, Wednesday, Thursday and Friday, May 8, 9, 10 and 11, 1888, commencing Tuesday at 11 A. M. Dr. J. Taft is chairman, and Dr. E. S. Talbot Secretary of the Section of Oral and Dental Surgery.

---

TALMAGE advertises for a text for his sermon before the dental convention. How about "The noise of the grinders is low"?—*Med. and Surg. Reporter.*

Where may that text be found? It certainly is not in the Bible. The old, shop-worn, back-number jest is not on the dentist this time.

---

THE CLIMATOLOGIST is the name of a new quarterly journal published in Washington and devoted, as its name indicates, to climatotherapy, epidemiology and preventive medicine. The first number is very attractive, and the journal gives promise of an abundant success.

---

DR. FORDYCE BARKER says that cancer is a disease of the most highly civilized, the most cultured, the worthy, and of localities that are the most salubrious, and that it is not hereditary.

Well, that opinion is consolatory, at least

---

MARRIED.—In New York City, February 21, 1888, Dr Sebert E. Davenport to Blanche Stevens. The happy parties will please accept the congratulations of THE INDEPENDENT PRACTITIONER.

---

OIL STAINS may be removed from marble by applying common clay saturated with benzine. If the oil has remained long enough it may have removed the polish, but the stain will disappear.

---

LAMBERT'S LISTERINE is one of the most perfect solvents for tannic acid that can be found. One ounce of it will dissolve half an ounce of the acid.—*South Western Med. Gazette.*

---

DR. L. D. SHEPARD, of Boston, is sadly afflicted in the death, by consumption, of a promising son of thirteen years. His many friends will sincerely condole with him.

---

AT THE LATE MEETING of the Mississippi Valley Dental Association, it was stated that Dr. J. Taft had not missed attendance since 1847, nor Dr. Geo. Watt since 1852.

---

DR. GEO. L. FIELD and wife, of Detroit, are spending some time in Florida and the south. Dr. and Mrs. A. P. Southwick, of Buffalo, are also in Florida.

---

THERE IS ALWAYS something pathetic in the calling of names by a weak writer. It gives him the kind of relief that women find in tears.

---

THE DENTAL COSMOS announces another enlargement. Henceforth each number will contain eighty pages.

---

THERE ARE one hundred and twenty medical colleges in full blast in the United States.

---

A DAUGHTER was born to Dr. B. L. Rhein, of Chicago, March 1, 1888.

